# Ischemic Optic Neuropathy: A Review of Current and Potential Future Pharmacotherapies

**DOI:** 10.3390/ph17101281

**Published:** 2024-09-27

**Authors:** Omar Badla, Beshr Abdulaziz Badla, Amr Almobayed, Carlos Mendoza, Krishna Kishor, Sanjoy K. Bhattacharya

**Affiliations:** 1 Department of Ophthalmology, Bascom Palmer Eye Institute, University of Miami Miller School Medicine, Miami, FL 33136, USAaxa4141@miami.edu (A.A.);; 2 Miami Integrative Metabolomics Research Center, Miami, FL 33136, USA; 3 College of Medicine, Mohammed Bin Rashid University of Medicine and Health Sciences, Dubai P.O. Box 505055, United Arab Emirates; beshr.badla@students.mbru.ac.ae; 4 Graduate Program in Molecular and Cellular Pharmacology, University of Miami Miller School of Medicine, Miami, FL 33136, USA

**Keywords:** ischemic optic neuropathy, ION, AION, AAION, NAAION, NAION, PION, management, pharmacotherapy

## Abstract

The treatment of arteritic anterior ischemic optic neuropathy (AAION), non-arteritic ischemic optic neuropathy (NAAION), and posterior ischemic optic neuropathy (PION) is a topic of ongoing research with mixed evidence on some pharmacotherapies and a need for more consensus. This manuscript provides an overview of these conditions’ current, potential future, and attempted pharmacotherapies. AAION’s current treatment regimen consists of high-dose steroids, with methotrexate, tocilizumab, and abatacept, being the most viable steroid-sparing therapy candidates. As for NAAION, the treatments being tried are vast, with mixed evidence supporting each modality. Similarly, despite the various treatment options explored, there still needs to be a universally effective therapy for PION. More research is needed to formulate an agreed-upon treatment regimen for these conditions.

## 1. Introduction

**Figure 1:**
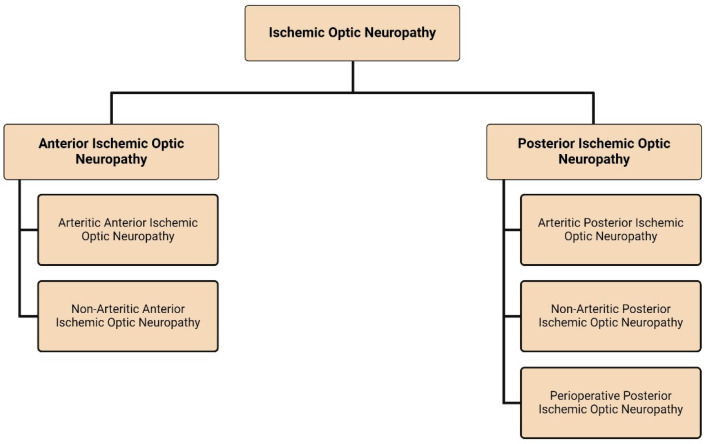
The subtypes of AION and PION. Created with Biorender.com.

Ischemic optic neuropathy (ION) encompasses a spectrum of diseases, each with its own causes, symptoms, and treatments [1]. Broadly, the condition can be divided into anterior (AION) and posterior (PION) optic neuropathies [1]. The subtypes of AION and PION differ and are detailed in Figure 1. Arteritic anterior ischemic optic neuropathy (AAION) accounts for 5–10% [2] of AION cases. AAION is essentially a manifestation of giant cell arteritis (GCA). However, other types of vasculitis, like systemic lupus erythematosus, polyarteritis nodosa, and herpes zoster, can also be a cause [2]. Non-arteritic anterior ischemic optic neuropathy (NAAION) is an important cause of vision loss in patients aged 50 and older [3]. While the exact pathogenesis remains elusive, it is hypothesized to be caused by decreased perfusion by the short posterior ciliary arteries (SPCAs) to the optic nerve head (ONH) leading to ischemia, edema, and compression of the optic nerve (ON) with subsequent retinal ganglion cell (RGC) death [3,4,5]. Vision loss in PION is due to ischemia of the posterior part of the ON. The optic disc looks normal during the acute phase because the site of the ischemia is farther back in the ON; with the advancement of the ischemic degeneration, descending optic atrophy will occur [6]. The following sections will delve into the current, potential future, and attempted pharmacotherapies for these conditions.

## 2. Anterior Ischemic Optic Neuropathy

### 2.1. Arteritic Anterior Ischemic Optic Neuropathy

The pathogenesis of AAION involves inflammation of the SPCAs leading to their thrombosis and infarction of the ONH [2]. Therefore, anti-inflammatory strategies are commonly applied to treat patients with AAION using pharmacotherapies such as corticosteroids, tocilizumab, methotrexate, abatacept, and tumor necrosis factor-alpha (TNF-α) inhibitors. Table 1 acts as a summary of the treatment strategies of AAION.

#### 2.1.1. Steroid Treatment Strategies

There are multiple treatment regimens utilizing corticosteroids, which all aim to reduce the inflammation in the vessels leading to AAION. They induce an anti-inflammatory effect by crossing into the cell nucleus and increasing the transcription of anti-inflammatory genes, thereby preventing further thrombosis in the inflamed arteries of the retina caused by GCA and reducing the resultant edema [7]. An acute attack of AAION is an ophthalmological emergency as blindness in the affected eye can rapidly develop and is an even bigger emergency in the case of bilateral AAION as this can lead to total blindness. This is why despite different corticosteroid regimens, it is essential in the treatment of AAION to administer a very large dose quickly upon presentation in patients with visual manifestations. Hayreh et al. described the following regimen: intravenous (IV) methylprednisolone (MP) (1 g/kg/day) administered for 3 days followed by at least 4–6 weeks of oral prednisolone at 1 mg/kg/day [8]. The dose is then only tapered guided by levels of erythrocyte sedimentation rate (ESR) and C-reactive protein (CRP) [2,8]. Tapering needs to be a slow delicate process, as flare-ups can occur. Recommendations for tapering are as follows: reducing the dose by 10 mg per month till 20 mg/day is reached, to then by 5 mg every month, and then by 1 mg per month until a dose of 10 mg/day is reached [9]. To highlight the approach of rheumatologists to GCA, the recommended regimen by the British Society for Rheumatology is as follows: in the absence of ischemic visual manifestations, an initial dose of 40–60 mg oral prednisolone daily, in the presence of ischemic visual manifestations, 500 mg^−1^g IV MP daily for up to 3 days before commencing oral prednisolone therapy [10]. The IV dosage is lower than that described before; however, the guidelines demonstrate that even rheumatological guidelines consider high-dose glucocorticoids necessary for the treatment of GCA with visual manifestations. The tapering process can take at least 1–2 years [9] or, as reported by Hayreh et al., a median of 48.7 months (95% CI: 34.6–71.4 months) [2]. Once the lowest dose for each individual patient is reached, it is considered from an ophthalmologist’s view that treatment to prevent lifelong risk of blindness has to also be lifelong [2,8,9]; while according to the British Society for Rheumatology, glucocorticoid dose should be tapered to zero over 12–18 months [10]. This highlights the difference between a rheumatologist’s approach and an ophthalmologist’s approach to GCA treatment, a gap that should be bridged as GCA patients can be primarily under a rheumatologist’s care, and preventing the significant morbidity for patients should be paramount. IV steroid induction in place of oral steroids leads to no reduction of total steroid dose by 1 year of treatment [11]; however, it leads to an increased likelihood of improvement in patients receiving IV steroid induction (40% vs. 13%) [12].

#### 2.1.2. Steroid Sparing Treatment Strategies

The previously mentioned long-term treatment of AAION as a manifestation of GCA involves a corticosteroid regimen over months and years. Well-known side effects of steroids include osteoporosis, steroid-induced myopathy, insulin resistance, hypertension, gastritis, and even psychiatric side effects such as anxiety and depression [13]. Strategies to decrease the exposure to corticosteroids, and therefore, their side effects, are described below.

- Tocilizumab

Tocilizumab is an anti-interleukin (IL)-6 receptor monoclonal antibody, which is an FDA-approved treatment for GCA. It is important, however, to recognize that treating GCA and preventing blindness due to GCA from AAION are two different entities. Randomized control trials looking at the effectiveness of both subcutaneous doses every week or every other week of tocilizumab as a steroid-sparing therapy for GCA found higher rates of sustained remission when combined with a 26-week course of prednisone (cumulative median dose = 1862 mg) at 52 weeks (56% and 53%, respectively) duration compared to a larger course of prednisone (cumulative median dose = 3818 mg) taken over 52 weeks at the same 52-week mark (18%) [14]. Other studies [15,16,17,18,19,20] have demonstrated similar steroid-sparing properties and effectiveness in the treatment of GCA; however, this effectiveness is studied over periods that do not demonstrate the life-long safety necessity of treating GCA to prevent blindness from AAION. This is further complicated by the side effect profile of long-term tocilizumab therapy such as cytopenias, upper respiratory tract infections, bronchitis, and liver toxicity [21]. This, at the same time, does not preclude the use of tocilizumab in the context of AAION. It could be used as a steroid-sparing therapy up until proven safety periods, as Unizony et al. demonstrated a decreased incidence of visual manifestations for GCA patients on tocilizumab [22] and as reported by Vionnet et al., as a salvage therapy after treatment failure in patients with corticosteroid-resistant progressive AAION [23].

- Methotrexate

Methotrexate has also been considered as a steroid-sparing strategy candidate. Its proposed mechanism in treating AAION is decreasing the inflammation through multiple mechanisms including repressing T-cell activation, down-regulating B cells, and its antifolate antimetabolite action that all act to suppress the immune system, and therefore, the inflammation in AAION [24]. The adverse effects of methotrexate include gastrointestinal manifestations, hepatotoxicity, bone marrow suppression, and teratogenicity [24]. For GCA, Mahr et al. found that methotrexate did decrease the exposure to corticosteroids and also reduced the risk of relapses when used as an adjunctive treatment [25,26]. Again, the treatment of GCA and preventing visual loss from it should be considered distinct; however, one can infer the effectiveness of decreasing relapses to directly decrease the disease activity leading to visual loss.

- Abatacept

Abatacept, a cytotoxic T-lymphocyte-associated protein 4 (CTLA-4) immunoglobulin, is another candidate for steroid-sparing strategies and has been in use for the treatment of other diseases with a known side effect profile. Abatacept has not been studied specifically in the prevention of long-term visual losses in AAION due to GCA; however, Langford et al. conducted a randomized, double-blind trial of abatacept for the treatment of GCA. In that trial, abatacept and glucocorticoids were started until the 12th week of treatment, when patients free of remission were randomized to keep receiving abatacept monthly until 12 months with a tapered glucocorticoid dose until the 28th week or a tapered glucocorticoid dose only until the 28th week. Relapse-free (defined in this study as the need to increase prednisone dose or restart prednisone after discontinuation) rates at 12 months in the abatacept-treated group were significantly higher at 48% compared to the prednisone-only group at 31% as well as a significantly longer median duration of remission (9.9 months vs. 3.9 months) [27]. They found no significant difference in adverse effects between the two groups, making abatacept a viable candidate for future investigation looking at long-term ocular manifestations of GCA and if an abatacept–corticosteroid regimen with lower steroid load is safe.

- TNF-α inhibitors

Another one of the biological treatments is TNF-α inhibitors, which are used in the treatment of multiple inflammatory and autoimmune disorders such as inflammatory bowel disease, psoriasis, rheumatoid arthritis, ankylosing spondylitis, and more [28]. In the case of GCA, however, they have not shown significant benefits or efficacy in its treatment [10,29].

- Prostaglandin E1

Prostaglandin E1 (PGE1) is a vasodilator whose effects can be theoretically used to help restore circulation and blood flow through the PCAs and prevent ischemia of the ONH. Adding PGE1 to the initial high-dose corticosteroid regimen has been attempted in a case report by Steigerwalt et al. who found improved and stable visual acuity (VA) in these two patients who remained stable on follow-up examinations [30]. This, however, is a small sample size, and larger studies are necessary to evaluate its true effects in AAION patients.

**Table 1:** Summary table of the discussed treatment strategies of AAION.

Pharmacotherapy	Design of Studies Analyzed	Year of Publication	Conclusions
Corticosteroids	Review [2].	2009	Treatment with a rapidly administered large dose of corticosteroids followed by a long tapering steroid regimen remains the mainstay of AAION treatment. There remains a gap between the length of corticosteroid regimens recommended by ophthalmologists and rheumatologists.
	Review [8].	2011	
	Review [9].	2021	
	Guideline Study/Review [10].	2020	
	Clinical Trial [11].	2000	
	Retrospective Study [12].	2001	
Tocilizumab	Clinical Trial [14].	2017	An FDA-approved for the treatment of GCA, tocilizumab has demonstrated effectiveness as a steroid-sparing therapy. Further studies must be conducted looking at tocilizumab in the context of AAION due to GCA past the currently studied timelines.
	Randomized, Double-Blind, Placebo-Controlled Trial [15].	2016	
	Retrospective Study [16].	2016	
	Case Series [17].	2011	
	Retrospective Study [18].	2015	
	Retrospective Study [19].	2012	
	Case Report [20].	2014	
	Retrospective Study [22].	2021	
	Review [23].	2017	
Methotrexate	Meta-Analysis [25].	2007	Methotrexate was reported to effectively reduce the risk of relapse and corticosteroid exposure in GCA patients. More should be studied about its role in the context of AAION and vision protection.
Abatacept	Randomized, Double-Blind Trial [27].	2018	Demonstrated lower remission rates and corticosteroid exposure in the treatment of GCA with a good adverse effect profile. Viable candidate for a future investigation looking at long-term ocular manifestations of GCA.
TNF-α inhibitors	Guideline Study/Review [10].	2020	Has not demonstrated significant benefits or efficacy in the treatment of GCA and, therefore, its ocular manifestations.
	Review [29].	2022	
Prostaglandin E1	Case Report [30].	2010	In the referenced case report, PGE1 was found to improve and stabilize visual acuity (VA) in two patients on follow-up. Larger studies are necessary to determine its efficacy.

### 2.2. Non-Arteritic Anterior Ischemic Optic Neuropathy

Currently, there is no widely accepted treatment for this NAAION, and management is limited to risk factor mitigation [3,4]. In the following sections, we will provide an overview of this condition’s current and potential future pharmacotherapies, ranging from pharmacotherapies tested in vitro to pharmacotherapies tested in humans (Table 2).

#### 2.2.1. Corticosteroids and Associated Pharmacotherapies

- Corticosteroids:

Corticosteroids are hypothesized to treat NAAION by reducing disc swelling and preventing free-radical-induced damage [31]. Experiments conducted on rat models of non-arteritic anterior ischemic optic neuropathy (rAION) have tested MP’s anti-inflammatory and neuroprotective efficacy on ONs and RGCs [5,32]. In two experiments, intraperitoneal MP was administered to experimental rats, and control rats received intraperitoneal phosphate-buffered saline (PBS) [5,32]. Both studies reported the neuroprotective effects of MP treatment. Specifically, the early administration of MP was associated with anti-inflammatory effects [5] and increased RGC survival and ON function [32]. However, in studies on human patients, a single-masked, randomized clinical trial assessing the effect of 500 mg of IV MP, given twice a day for three days and followed by 2 weeks of oral prednisolone, found that this regimen does not improve the final visual and structural outcome in NAAION patients compared to controls [33]. Likewise, a prospective interventional comparative case series tested the same dose of IV MP followed by oral dosing. This study concluded that this regimen did not produce a beneficial effect compared to controls [34]. A randomized, double-blind, controlled trial conducted on nondiabetic NAAION patients evaluated the role of oral corticosteroids in treating this condition [31]. Nineteen patients were given oral prednisolone (80 mg) for 2 weeks, tapered to 70 mg, 60 mg, then by 5 mg at five-day intervals until completion. Another 19 patients were on placebo. The primary outcome measures included best-corrected visual acuity (BCVA), changes in the retinal nerve fiber layer (RNFL) on optical coherence tomography (OCT), and visual evoked response (VER) [31]. In the steroid group, there were statistically significant improvements in VER parameters and BCVA coupled with a quicker resolution of disc edema on OCT on a 1-month follow-up. Despite that, these improvements were deemed clinically insignificant, as there was no significant difference in VA improvement in the steroid group compared to controls at 6 months [31]. Similarly, another randomized controlled trial evaluated the use of 75 mg of oral prednisolone on NAAION patients within five days of diagnosis and recommended against its use [35]. A retrospective analysis compared the visual outcomes of four patient groups: a control group, a group on oral prednisone, a group on 250 units of MP followed by oral prednisone, and a group on 500 units of MP followed by oral prednisone [3]. This study noted a statistically significant improvement in BCVA in all steroid groups compared to baseline. However, it reported no significant differences in the final visual outcomes when comparing the four groups [3]. One notable finding highlighted in this study is the short-term improvement in BCVA in patients receiving 500 units of MP during the 7- to 14-day follow-up period, compared to baseline [3]. Lastly, a systematic review and meta-analysis conducted on studies dating until the 10th of June 2019 assessed different therapies for NAAION and concluded that steroids do not significantly improve VA and visual field (VF) [4]. While experimental studies on the neuroprotective effects of MP on rAION models proved effective, studies on human patients have failed to showcase a clinically significant improvement in final VA compared to controls.

- Triamcinolone:

Intravitreal triamcinolone (IVTA) has been tried for its anti-inflammatory effects and to avoid the systemic side effects associated with IV or oral administration of corticosteroids [36]. Experiments conducted on rAION models tested the efficacy of this treatment modality. One experiment utilized three groups of female Wistar rats, with group one receiving no treatment, group two receiving 56 μg of IVTA, and group three receiving intravitreal saline [37]. The treatment was administered within 10 min of rAION induction, and the rats were euthanized 30 days later, after which the RGC nuclei were counted [37]. This study found no significant difference in RGC count across the three groups and concluded that this treatment was not beneficial [37]. Another study utilized the rAION model but had three experimental groups that received 0.32 mg/2 μL of IVTA after 1, 7, and 14 days of rAION induction, respectively [38]. The control group received PBS. The results revealed that using ITVA within a week post-induction leads to increased RGC survival, visual evoked potential (VEP) electrophysiological amplitude, and decreased immune cell infiltration of the ON [38]. The difference in conclusions between the two studies could be due to the different dosing scales and outcome measurement techniques. As for human studies, a systematic review and meta-analysis evaluated the use of IVTA, and while the treatment led to an improvement in VA and VF, neither of the two studies were trials and had a relatively small number of cases [4].

#### 2.2.2. Neuroprotective Agents

- EPO:

Erythropoietin (EPO) is expressed by various tissues, including neurons and glial cells, and has been tested for NAAION for its neuroprotective in vitro effects in neuronal injury animal models [34]. In a prospective interventional comparative case series, IV recombinant human EPO was administered with systemic IV corticosteroids to patients with NAAION within 14 days of diagnosis [34]. This combination had no beneficial effect compared to the control group or the group that received steroids alone [34]. The subsequent study performed by the same group of researchers evaluated IV EPO independently and did so for cases within five days of diagnosis to study the drug’s effect at a narrower therapeutic window [35]. In this randomized control trial, the EPO group received 100,00 units twice daily for three days. At the 6-month follow-up, the EPO group had a statistically significant difference in improvement in BCVA when compared to the steroid group and controls [35]. Moreover, the patients in the EPO group experienced a lesser decrease in the thickness of the peripapillary RNFL compared to the two other groups [35]. These findings suggest that IV EPO exerts a functional and structural neuroprotective effect on the ONs of NAAION patients if used within five days of diagnosis [35].

- G-CSF:

Granulocyte colony-stimulating factor (G-CSF) is a hematopoietic growth factor used to treat neutropenia [39]. Recently, studies have delved into its neuroprotective in Alzheimer’s and Parkinson’s diseases [39]. The G-CSF receptor is present in RGCs and other neural cells [39], which has led to its application in rAION models to test its efficacy in treating NAAION. Experiments involving this rat model have demonstrated G-CSF’s anti-inflammatory and antiapoptotic effects by preserving RGC density, decreasing ED-1-positive cell infiltration [40], and reducing pro-inflammatory cytokine expression [41]. Moreover, combining G-CSF with meloxicam, an anti-inflammatory cyclooxygenase two inhibitor, resulted in similar anti-inflammatory and antiapoptotic effects in the rAION as mentioned above, with the additional effect of decreasing GCS-F-associated leukocytosis [42]. More recently, it was discovered that G-CSF exerts one of its neuroprotective antiapoptotic effects by modulating the TAF9-P53-TRIAP1-CASP3 pathway [39]. In a prospective interventional case series pilot study, an intravitreal G-CSF injection was administered at 60 µg per 0.1 mL within 2 weeks of NAAION onset [43]. The results of this study revealed an improvement in BCVA at the 1-month follow-up, but the final BCVA showed no significant improvement compared to the baseline [43]. The study concluded that the injection is safe, but the effect lasts a month and then declines [43]. The next step would be to perform a controlled trial with more cases and potentially test the use of multiple injections to maintain the effect.

- Citicoline:

Citicoline has been considered for NAAION due to its neuroprotective effects [44]. The mechanism by which the drug exerts its neuroprotective effect is by targeting the undamaged axons [44]. A randomized pilot study enrolled 36 NAAION patients and 20 age-matched controls to test the effect of a 500 mg/day oral solution of citicoline on VA, pattern electroretinogram (PERG), VEP, Humphrey 24-2 visual field mean deviation (HFA MD), and RNFL thickness [45]. The results of this study revealed a significant difference in all the criteria mentioned above between the groups at 6 months; therefore, the conclusion is that citicoline exerts a neuro-enhancing and neuroprotective effect in NAAION patients [45]. These results are promising but need to be supported by a study with a larger cohort and long-term follow-ups to assess the longevity of the drug’s effect.

- Trabodenoson:

Trabodenoson has been theorized to treat NAAION by activating the A1 receptor (A1R), an adenosine receptor that exerts a neuroprotective effect [46]. One study tested the efficacy of this drug in a topical daily 3% formulation on a rodent NAION model (rNAION). The drug was started 3 days before and 21 days after ischemia induction [46]. This formulation successfully reduced ON edema and preserved RGC count and ON axons in rNAION vs. control rodents [46]. These results are promising and showcase the neuroprotective effects of topical trabodenoson. However, this formulation must be evaluated in human NAAION patients before it can be considered for this condition.

- Vincamine:

Vincamine is an extract from the Apocynaceae Vinca plant, and it has been tested on a rAION model for its potential neuroprotective qualities [47]. This study found that intragastric Vincamine has an antiapoptotic effect on RGCs of rAION rodents. The proposed mechanism involved in its neuroprotective role is the PI3K/Akt/eNOS pathway. This study was limited by the small number of rodents per group, the short duration of the follow-up, and the lack of pAKT/eNOS testing in the 4 weeks leading up to the first measurement [47]. In a study conducted on 27 human NAAION patients and 15 age-matched controls, vincamine was given to the patients in the treatment group, and both groups received steroid pulse therapy and neurotrophic treatment [48]. The treatment group had a statistically significant larger improvement in mean deviation of the visual field and RNFL and ganglion cell complex thickness [48]. These results are promising but need to be verified with a trial containing more patients.

- CNTF:

Ciliary-derived neurotrophic factor (CNTF) is comparable to IL-6 in its antiapoptotic effect on RGCs in response to injury [49]. Hence, an experimental study tested its potential neuroprotective effect in an rAION model [50]. This study evaluated the effect of an intravitreal injection of 0.75 μg one day after rAION induction, with control mice receiving a sham injection. Fifteen days after ischemic induction, the rodents were euthanized, and their RGCs were counted by identifying cyan fluorescent protein (CFP) cells using stereology in a flat-mounted retina [50]. Results revealed a higher number of CFP-positive cells in CNTF-treated rodents than in sham-treated rodents and a lower decrease in CFP-positive cell density in experimental vs. control RGC layers [50]. These results are promising and showcase the neuroprotective effect of this drug. However, for this effect to apply to NAAION patients, further studies need to be performed to showcase the sustainability of this effect over a more extended follow-up period using tests that can observe visual function over that period, such as PERG. Moreover, human studies need to be performed too down the line.

- BDNF/LM22A-4:

Brain-derived neurotrophic factor (BDNF) exerts a neurotrophic effect that enhances RGC survival by activating tropomyosin-related kinase B (TrkB) receptors [51]. However, BDNF poses some possible limitations, including infection, inflammation, and poor blood–brain barrier penetration [51]. Therefore, safer activation of the TrkB receptors can be achieved by using (N,N’,N’-tris [2-hydroxyethyl])-1,3,5-benzene tricarboxamide (LM22A-4), a partial agonist specific to the TrkB receptors [51]. The effects of LM22A-4 were tested in this study in vitro by assessing its effects on the survival of immunopanned RGC cultures and in vitro by assessing the effect of its intravitreal injection and 3-week systemic administration on RGC density and serial OCTs of a murine AION model [51]. The results revealed a significant difference in all measured parameters, which indicates a neuroprotective and beneficial effect for NAAION [51]. Another tested application for BDNF in the treatment of NAAION is its incorporation with bone marrow-derived stem cells and their combined effect in the affected eyes of the rAION model [52]. BDNF improved the quantity and quality of engrafted stem cells and the neuroglial differentiation of the stem cells within the ischemic retina [52]. Overall, BDNF and LM22A-4 have promising beneficial direct and indirect effects on animal models of NAAION. However, more extensive animal studies need to be conducted with extended follow-up periods to monitor this treatment’s long-term effects before advancing to human studies.

- Memantine:

Memantine is an NMDA receptor antagonist that blocks glutamate-mediated neurotoxicity in RGCs [4]. A systematic review and meta-analysis found that memantine improved VA when analyzed as a continuous variable but not as a categorical variable. Additionally, the drug was found not to improve VF [4].

- Minocycline:

Minocycline, a tetracycline derivative, has been shown to be neuroprotective in hypoxic and ischemic CNS models [53]. This effect can be explained by its inflammation-modulatory effect [53]. Additionally, it has been reported that it has neuroprotective effects in animal glaucomatous and traumatic optic neuropathies [53]. These findings prompted the trial of daily intraperitoneal minocycline (33 mg/kg) in an rNAION model [53]. While this study’s results highlighted the immunomodulatory effect of minocycline, the RGC survival did not improve with the administration of minocycline compared to controls [53]. Therefore, compared to previously mentioned neuroprotective drugs, minocycline does not show promise in its ability to preserve RGCs.

- Butylidenephthalide:

Butylidenephthalide (BP) is a component of Angelica, a Chinese medicine with many applications [54]. Research on BP has found it to be effective for its antitumor, anti-inflammatory, and neuroprotective qualities [54]. These qualities made BP a potentially valuable agent for an ischemic injury model; therefore, its effects on RGC survival, visual function, and inflammatory response inhibition were tested in the rAION model [54]. For this purpose, three rAION groups were used: sham, PBS-treated, and BP-treated. The BP group received 10 mg/kg of the drug intraperitoneally for seven consecutive days, after which different parameters were assessed [54]. BP was found to increase RGC survival rates, prevent visual loss on flash visual evoked potential (FVEP) stimulation, improve optic disc edema, preserve RNFL thickness on OCT, and decrease macrophage infiltration into the ON compared to controls [54]. One of the mechanisms by which BP is thought to diminish the inflammatory response is through the NF-κB pathway [54]. While the early experimental results are promising, it is essential to follow the experimental evidence with human studies to validate the effect of this drug in NAAION patients.

- Bardoxolone Methyl and Omaveloxolone:

Similarly, bardoxolone methyl (RTA 402) and omaveloxolone (RTA 408), two synthetic oleanane triterpenoids, also inhibit the NF-κB pathway, which accounts for their anti-inflammatory and antioxidative properties [55]. These properties prompted the trial of RTA 402 and RTA 408 for NAAION in an rAION model [55]. The study included six groups: an rAION + RTA 402 (20 mg/kg) group, an rAION + RTA 402 (40 mg/kg) group, an rAION + RTA 408 (10 mg/kg) group, an rAION + RTA 408 (20 mg/kg), an rAION + PBS group, and a sham group [55]. Each group includes 18 rodents, six of which will be tested using FG retrograde labeling, six will undergo OCT, VEP, terminal deoxynucleotidyl transferase- (TdT-) dUTP nick end labeling (TUNEL), and IHC, and six will undergo immunoblotting analysis [55]. The results for RTA 402 revealed anti-apoptotic, anti-inflammatory, antioxidative stress, and myelin-preserving effects on RGCs coupled with a visual function-preserving effect [55]. These results were not found with RTA 408 [55]. The evidence points towards RTA 402’s ability to modulate the Nrf2 and nuclear transcription factor nuclear factor kappa beta (NFκB) pathways as the reason for its RGC protective effect [55]. Overall, the results show that RTA 402 is a potential treatment for NAAION, but more comprehensive studies involving human patients are needed.

- Prostaglandin J₂ and MAGL/COX inhibitors:

Prostaglandin J2 (PGJ2) has been shown in rNAION models to preserve RGCs, RGC function, and ON function compared to controls injected with PBS [56]. PGJ2 antagonizes NFκB and activates nuclear factor peroxisomal proliferator-activated receptor-gamma (PPARγ), thus decreasing inflammation and neuronal apoptosis, respectively [56]. Miller et al. assessed the toxicity and efficacy of IV and intravitreal (IVT) PGJ2 in the treatment of a nonhuman primate model of non-arteritic anterior ischemic optic neuropathy (pNAAION). They found a significant reduction in histological damage as measured by axon counts (*p* = 0.05) and preserved electrophysiological function as measured by VEP and PERG (*p* = 0.03) in pNAAION treated with a single dose of IVT PGJ2 [56]. As for toxicity, no evidence of persistent ocular toxicity was found at the dose of IVT PGJ2 chosen for efficacy experiments (50 μg); however, at higher doses, transient ON function loss was observed, which resolved within a week. This toxicity must be considered for any future human studies evaluating the safety and efficacy of IVT PGJ2 in human NAAION. Mehrabian et al. investigated the addition of a monoacylglycerol lipase (MAGL) inhibitor KML29 to decrease the production of arachidonic acid (AA) and, therefore, downstream inflammation. In addition to KML29, they also investigated the addition of downstream inhibition of inflammation using cyclooxygenase 1/2(COX1/2) inhibitor meloxicam. They however found that neither KML29 nor meloxicam improved ONH edema or RGC survival in combination with PGJ2 better than reported PGJ2 alone [57]. They also reported that meloxicam and KML29 alone improve RGC survival compared to the vehicle alone [57].

- QPI-1007

QPI-1007 is a caspase-2 inhibitor and was considered a potential treatment for NAAION as it would inhibit apoptosis, increase RGC survivability, and therefore act as a neuroprotector. A phase 2/3 RCT of the efficacy of intravitreal QPI-1007, measuring the primary outcome of VA loss as compared to the sham intravitreal injection group in the treatment of human NAAION, was based on results of a phase 1 trial where a small sample of patients had no serious adverse effects and better preservation of VA compared to ION historical controls [58]. The phase I trial compared its small cohorts to bigger cohorts in historical controls, making the comparison weaker. It also had multiple investigators at 28 sites making it more difficult to ensure the validity of the results. Nevertheless, the phase 2/3 RCT was conducted and then terminated as the results were not positive. From the data released in the EU clinical trials register, no significant difference in the primary outcome between any of the four treatment regimens with QPI-1007 as compared to the control group was found. This result highlights the difficulty of translating in vitro or rat model results into real-world treatments but also the importance of trials taking place to test potential treatments [59].

- RPh201

RPh201 is an extract from gum mastic that has been studied in both phase 1 and phase 2 studies in the treatment of NAAION. Rath et al. claimed that RPh201 induces neuronal differentiation, synaptogenesis, immunomodulation, and neuroprotective effects in vitro and in vivo in unpublished data [60]. Based on this, a phase 1 trial was conducted, which established the safety of subcutaneous RPh201 in healthy volunteers [61]. A phase 2a single-site, prospective, randomized, placebo-controlled, double-masked trial in 20 patients was then conducted to assess the safety and changes in visual function and structure in patients diagnosed with NAAION for at least 6 months and a maximum of 3 years. They found RPh201 to be safe and a statistically insignificant improvement in BCVA from baseline as compared to the placebo group (14.8 ± 15.8 letters for RPh201 and 6.6 ± 15.3 for placebo, *p* = 0.27) [60]. The sample size is small, no statistically significant improvement was found, and the rationale for treatment after 6 months of diagnosis is questionable due to the irreversible nerve damage that would have occurred by then [62]. Nevertheless, a phase 3 double-masked clinical study evaluating the efficacy and safety of RPh201 in patients with previous NAAION was started in 2018 and completed in 2020, with results unpublished on ClinicalTrials.gov (NCT03547206). With unpublished results, no conclusion can definitely be drawn about the efficacy of RPh201, and it remains to be seen if the results will be published or not.

- Vitamin B3

Vitamin B3, or niacin, acts as a precursor to the coenzymes nicotinamide adenine dinucleotide (NAD+) and nicotinamide adenine dinucleotide phosphate (NADP+), which are needed in the synthesis of ATP. Vitamin B3 has been reported to prevent RGC and axonal loss in a glaucoma rat model [63]. Chen et al. looked at the neuroprotective properties of vitamin B3 in a rat model of NAAION and found that compared to the saline-treated rats, B3-supplemented rats had significantly higher P1-N2 flash visual evoked potential (FVEP) (62.9 ± 10.37 μV vs. 32.84 ± 7.62 μV), significantly higher RGC density (1086.7 ± 133.4/mm^2^ vs. 647.2 ± 98.5/mm^2^), and a significant reduction in oxidative damage [63]. Niacin is a commonly available vitamin supplement with proven safety, so trials in humans supplementing existing treatment regimens are an option to further understand its role in neuroprotection in NAAION patients.

- M01, a HECT domain-E3 ubiquitin ligase inhibitor:

Based on the studies showing the role of E3 ligase activity suppression in prolonging the survival of axons following ON injury in rNAION models [64], Chien et al. investigated the role of M01, a homologous E6-associated protein carboxyl terminus (HECT) domain-E3 ubiquitin ligase inhibitor in neuroprotection of RGCs in the ON and retina of rNAION models. They elucidated that M01 upregulated nuclear factor erythroid 2-related factor 2 (NRF2), a transcription factor involved in the regulation of antioxidant proteins, which subsequently decreased levels of Thioredoxin interacting protein (TXNIP), an oxidative stress mediator, and NLR family pyrin domain containing 3 (NLRP3), an inflammasome. These actions lead to a decrease in TUNEL-positive cells, which indicate apoptotic RGCs, post NAAION induction in the M01 treated group (7.1 ± 3.9/HPF) compared to the saline group (15.3 ± 4.5/HPF) [64]. They also found decreased edema, inflammatory factors, demyelination, and an increase in M2-subtype microglial polarization. These findings present the modulation of HECT domain-E3 ubiquitin ligase pathways as a new approach toward the treatment of NAAION that needs to be further investigated to fully identify its efficacy and safety.

- Brimonidine

Brimonidine tartrate is an alpha-adrenergic agonist, which has been studied in humans as a treatment for NAAION. Brimonidine had promising reports of neuroprotective ability in animal studies [65] and is safe for human use, so it was a good candidate for human studies. Despite its promising results in animal studies, topical brimonidine has been demonstrated to not improve VA or VF significantly in human trials [4,49,66,67,68]. This result highlights the need for human trials to fully evaluate a candidate drug that was found to be beneficial in animal trials.

- Progesterone:

The hormone progesterone has been demonstrated in modes of central nervous system injury and cerebral ischemia models to provide neuroprotection and reduce infarct volume leading to functional recovery, respectively [69]. On this basis, Allen et al. aimed to investigate the role of progesterone treatment in rat models of NAAION and of middle cerebral artery occlusion (MCAO). These represent two different models of ocular ischemia, and the results were different for either model. In the rAION model, progesterone showed no effect on VEP reduction or RGC loss; however, in the MCAO model, progesterone reduced electroretinogram deficits and RGC loss [69]. While the results are not promising in the context of NAAION, progesterone remains a target for future research surrounding neuroprotection in other contexts.

- PLGA-Icariin

Sourced from the Epimedium spp., icariin is a flavonoid glucoside which is a natural product that has been found to have anti-inflammatory properties [70]. Desai and Wen et al. studied the long-term therapeutic effects of icariin in a rat NAAION model. Icariin’s short half-life was prolonged with the use of poly(lactide-co-glycolide) (PLGA) microspheres to have a longer-lasting release of intravitreally administrated icariin. The anti-inflammatory effect was determined by them to be through induction of endogenous G-CSF expression, which activated the non-canonical NF-kB signaling pathway by promoting phosphorylation of inhibitor of nuclear factor kappa-B kinase subunit beta (IKK-β) [70]. This anti-inflammatory effect of icariin was found to significantly reduce ON edema, macrophage infiltration, and increased RGC density as well as P1-N2 amplitude evidenced by FVEP measurements at day 28 after rAION induction [70]. The elucidated pathway and the neuroprotective results of this study provide possible future targets and a basis for intravitreal icariin to be further studied in the setting of human studies.

- Puerarin:

Puerarin is a bioactive ingredient extracted from the root of Pueraria lobata and has been found to have vasodilatory, neuroprotective, anti-ischemic, and anti-inflammatory activity through multiple mechanisms in animal models [71]. MANN Le et al. studied these effects of puerarin in a rat model of NAAION and measured the FVEP, RGCs, and inflammatory response. They report a significant 2.3-fold higher P1-N2 amplitude in FVEP analysis, a 1.6-fold higher RGC density, and 2.8-fold lower apoptotic RGCs in the puerarin-treated group than the saline group [72]. These promising results offer a base for targeting the specific mechanism that acts as neuroprotection or the usage of puerarin itself in future studies for the treatment of NAAION.

- miR-124:

The microRNA miR-124 was found in experimental lab models by Chen et al. to have neuroprotective effects on RGCs due to increased survivability and an improved visual function measured by FVEP by altering gene expression towards upregulating anti-inflammatory reactions through their capacity to activate anti-inflammatory M2 macrophages [73]. This novel approach requires human testing to be evaluated as a treatment in patients suffering from NAAION.

#### 2.2.3. Stem Cell Pharmacotherapies

- Mesenchymal Stem Cells:

Mesenchymal stem cells (MSCs) have applications in various research and therapeutic settings due to their immunomodulatory, tissue-regenerative, and ischemic tissue repair effects [74,75]. An experimental study assessed the safety of MSCs by injecting human Wharton’s jelly intravitreally into a standard set of Wistar rats [74]. The injections led to retinal venous congestion, damage, inflammation, and impaired visual function [74]. That being said, a recent prospective, non-randomized phase II study conducted on five NAAION patients ascertained that the treatment was safe and well tolerated except for the development of an epiretinal membrane in one patient [76]. Four out of five patients experienced visual improvement. Additionally, the P100 VEP amplitudes of three patients improved [76]. These findings need to be supported by larger studies.

- Mesenchymal Stem Cell Exosome:

Due to the potential limitations associated with MSC use, such as the ones discussed above, other MSC-derived treatments are being considered. One of these treatments is MSC-exosome, which has emerged as a promising therapy in managing various ischemic and neurodegenerative diseases and is more stable and less immunogenic than MSCs [75]. MSC-exosome has been theorized to treat NAAION by promoting neural plasticity and angiogenesis and reducing inflammation, immunity, oxidative stress, and apoptosis [75]. This modality has yet to be tested on any NAAION animal models, which is a primary step that needs to be taken to assess its safety and efficacy.

- Mesenchymal Stem Cell-Derived Medium:

Another MSC-derived treatment that has been considered for treating NAAION is MSC-derived conditioned medium (MDCM) [74]. This treatment incorporates soluble molecules derived from MSCs that retain MSC’s advantageous effects while limiting their unwanted side effects [74]. The efficacy of MDCM was assessed in the rAION model and was found to preserve visual function and RGC density and reduce inflammation in the ON [74]. These findings are promising but need to be supported by human studies.

#### 2.2.4. Anti-VEGF Pharmacotherapies

- Anti-VEGF:

This class of drugs can theoretically treat NAAION by decreasing the vasogenic edema associated with the condition by decreasing microvascular permeability [49,77]. This section will discuss the current evidence on three anti-vascular endothelial growth factor (VEGF) drugs.

- Bevacizumab:

A prospective trial tested intravitreal bevacizumab for NAAION and found no advantage for the drug compared to controls [78]. Additionally, a study reported that NAAION manifested in several patients who were administered intravitreal injections of an anti-VEGF treatment for age-related macular degeneration [79]. Only two case reports have noted the beneficial effect of intravitreal bevacizumab on NAAION. The first case report indicated that the treatment led to a rapid and significant resolution of the optic disc edema in NAAION [80]. The second case report evaluated the use of intravitreal bevacizumab in a case of NAAION with macular edema and subretinal fluid and reported a positive visual outcome [81]. As discussed, the evidence for this drug has been mixed, but the larger studies indicate that bevacizumab is ineffective for NAAION.

- Ranibizumab:

Intravitreal ranibizumab is another anti-VEGF agent that has been tried for NAAION, primarily in animal models. Studies in rAION and primate non-arteritic anterior ischemic optic neuropathy (pNAION) models have reported that intravitreal ranibizumab is ineffective for NAAION in these models [82,83].

- Aflibercept:

Conversely, a case report assessed the effect of a single intravitreal aflibercept, another anti-VEGF agent, and found that a 2 mg injection a day after the onset of NAAION significantly increased VA within the first week after the injection, reduced mean RNFL thickness by 22.7 μm, and improved VF at the 3-month follow-up [84]. Similarly, a retrospective trial assessed the effect of an intravitreal aflibercept injection of 2 mg/0.05 mL in 15 cases compared to 10 conservatively treated cases [85]. The results point towards a potential role for this treatment modality in acute NAAION in improving disc edema and VA, which need to be verified by conducting large-scale studies [85].

#### 2.2.5. Anti-Parkinson Pharmacotherapies

- Levodopa/Carbidopa:

Levodopa and Carbidopa act synergistically to increase dopamine levels in the central nervous system, which is said to have neuromodulatory and neuroprotective effects [4]. The effect of this combination has been tested for NAAION patients in multiple studies [4]. There have been conflicting results regarding the efficacy of treatment modality for NAAION. A retrospective study found that central VA improves if levodopa is used within 15 days of disease onset, which correlates with a maculopapular RGC-sparing effect [86]. Another retrospective study found that levodopa and carbidopa together led to an improvement in VA in NAAION patients with a VA of 20/40 or worse [87]. That being said, the 18 patients in the control group showed no improvement, which calls selection bias into question [4,87]. A randomized controlled trial of the same combination was performed on NAAION patients with long-standing vision impairment, and improvement was found in VA. However, the visual improvement could also be accounted for by the spontaneous improvement as NAAION resolved [4,88]. Additionally, another randomized placebo-controlled trial that used levodopa and carbidopa found the combination ineffective in correcting long-standing visual loss [89]. Overall, there seems to be contradicting evidence; therefore, more studies must be performed to support one conclusion.

#### 2.2.6. Blood-Associated Pharmacotherapies

- Aspirin:

Aspirin was attempted for the treatment and prevention of NAAION due to the condition’s association with vascular risk factors [49]. As a treatment option, aspirin was ineffective at improving VA and field outcomes in a retrospective study [49]. Aspirin was also assessed as a tool for preventing NAAION in the second eye with mixed results [49,79]. That being said, the largest retrospective studies indicate no long-term benefit to using aspirin in preventing NAAION in the second eye [49,79,90]. Despite the lack of evidence supporting its use in the prevention of NAAION in the second eye, experts still recommend aspirin after an episode of NAAION for its preventative effect of lowering stroke and myocardial infarction risk in this vasculopathic patient population [79].

- Platelet Rich Plasma:

Platelet-rich plasma (PRP) has also been attempted to treat NAAION [91]. In a prospective nonrandomized controlled trial, 12 NAAION patients received two tenon capsule injections of PRP and were compared to 13 control patients. Both groups received basic treatment with IV iodine hydrobromide and butylphthalide-sodium for 10 consecutive days. The two PRP injections were administered on days 1 and 10 of the basic treatment [91]. This trial showed that the PRP patient group’s BCVA improved between days 1 and 30. However, there was no significant difference between the PRP and control groups for the same parameters [91].

- Heparin-Induced Extracorporeal LDL/Fibrinogen Precipitation:

Heparin-induced extracorporeal LDL/fibrinogen precipitation (HELP) decreases plasma viscosity and improves microcirculation [4]. When analyzed in a systematic review and meta-analysis, HELP did not improve VA as a categorical variable [4].

- Anticoagulants:

Heparin and warfarin have shown no benefit in patients with NAAION [4].

#### 2.2.7. Miscellaneous Pharmacotherapies

- A Multivitamin, Mineral, Carotenoid, and Antioxidant Supplement Regimen:

Vega et al. conducted a retrospective case series study looking at the effect of a multivitamin, mineral, carotenoid, and antioxidant supplement regimen (including Vit A, B1, B2, B3, B6, B12, C + Zinc, selenium, manganese + lutein, zeaxanthin + glutathione, flavonoids, coenzyme Q10, with the stroke patients receiving aspirin) on VF measurements in 48 patients diagnosed with retinal vascular disease including NAAION, RAO, and homonymous hemaniopa or quadrantopia following stroke. [92]. In the NAAION patients, their visual field index (VFI) progressed at a rate of +11.5 ± 15% per year for the NAAION patients (n = 18, *p* < 0.0001), with most improvement occurring after 2 months of follow-up, meaning improvement is more likely due to supplementation than spontaneous resolution as part of natural disease history [92]. However, this case series has a small sample size with no comparison group, and the time of onset of visual loss was not known for each patient. There is also no way of knowing which of these supplements is contributing to the improvement. The improvements, however, are significant, and further studies evaluating such treatment regimens must be evaluated in randomized, double-blind, controlled, prospective clinical studies to fully support the use of these regimens in NAAION patients.

- 4-PBA:
- 4-phenylbutyric acid (4-PBA) is a chemical chaperone that has been studied in the treatment of cystic fibrosis, liver injury, and animal models of vision loss including glaucoma [93]. The unfolded protein response pathway is used by cells to control the endoplasmic reticulum (ER) and, in the case of cellular insult, to initially act as a defense line activating pro-survival pathways. However, after prolonged endoplasmic reticulum stress, pro-apoptotic pathways are upregulated, and this leads to cell death. Kumar et al. aimed to use intraperitoneally administered 4-BPA to reduce this ER stress and therefore preserve cell survivability in a mouse model of NAAION [93]. 4-PBA-treated NAAION eyes had a significant 22% higher number of RGC, and a significantly higher (5-μm) ganglion cell complex thickness on OCT imaging after induction of NAAION compared to the saline-treated group [93]. These results highlight the unfolded protein response pathway and decreasing ER stress using 4-PBA as valid therapeutic target candidates to be evaluated in human studies and serve in the treatment of NAAION.
- Endothelin Receptor Antagonists: Bosentan:
- Endothelin, a vasoconstrictive peptide released by both endothelial cells and vascular smooth muscle, is strongly implicated in cardiovascular disorders and in obstructive sleep apnea, a condition present in up to 70–85% of patients with NAAION [94]. Studies looking at blocking the effects of endothelin with endothelin receptor antagonist bosentan have found that it increased retinal blood flow at the ONH in both healthy and glaucoma patients [94]. For these reasons, Chiquet et al. considered bosentan a good candidate to target the vasoconstriction caused by endothelin in the acute phase of NAAION and are conducting a multicenter randomized controlled trial looking at change in VF, VA, quality of life, and macular ganglion cell layer thickness. As of 1 July 2024, the results of the clinical trial have not been published, but it remains a promising treatment option for NAAION, which should be followed up.
- Omega-3 Polyunsaturated Fatty Acids:
- Omega-3 polyunsaturated fatty acids (ω-3 PUFAs) are a common food supplement demonstrated to modulate different signaling pathways and inhibit both inflammation and cell apoptosis in multiple models [95,96]. This was studied by Georgiou et al. in rat models of AION. The rats were given ω-3 PUFAs by gavage for 10 days and compared to those given saline. Rats receiving the ω-3 PUFAs were then found to have higher RGC densities, higher amplitudes of FVEP, lower numbers of apoptotic cells in the RGC layer, reduced macrophage recruitment at the ON, and increased M2 macrophage anti-inflammatory markers than in the saline group [97]. This is yet to be evaluated in humans, and further experiments need to be performed to elucidate its uses in NAAION.
- Bioengineered Algae Oil:
- ω-3 PUFAs can contain different ratios of docosahexaenoic acid (DHA) to eicosapentaenoic acid (EPA), and with the background that one study showed that pure DHA or a combination containing more DHA than EPA promoted more expression of neurotrophins and their receptors in neuron cell lines [98], Huang et al. investigated the effects of algae oil from bioengineered marine microalgae Schizochytrium sp., which is a DHA-rich ω-3 PUFA in a model of rAION [99]. Huang et al. found significantly higher FVEP and density of RGCs in the algae oil-treated group [99]. In future human studies, the importance of DHA/EPA ratios in ω-3 PUFAs should be investigated as different ratios could play a role in treatment effectiveness.
- P-Selectin:
- P-selectin plays a role in the recruitment of leukocytes to platelet aggregates and in inflammatory leukocyte extravasations [100]; therefore, targeting this pathway could decrease the number of ischemic injuries in NAAION patients. Kapupara et al. investigated soluble recombinant P-selectin immunoglobulin G chimeric fusion protein in rat AION models and demonstrated an increased RGC survival rate through stabilization of the blood–ON barrier and increased Nrf2 transcription factors levels and activating its signaling pathway [100]. This target needs to be further evaluated in human experiments to assess its efficacy in NAAION patients.
- Anti-Nogo Antibody:
- Nogo-A is an inhibitory protein in the central nervous system that prevents the continued expansion of neurons at the end of development [101]. Johnson et al. investigated the role of anti-NOGO receptor monoclonal antibody 11C7mAb in a rat model of non-arteritic anterior ischemic optic neuropathy and found a higher rate of FVEP preservation and a reduction in microglia, extrinsic macrophages with axon sparing, decreased extracellular debris, and less myelin damage in those receiving the antibody versus the group receiving the vehicle only [101]. Further human studies are required to evaluate 11C7mAb as a treatment for NAAION.

#### 2.2.8. Future Pharmacotherapy Study Targets

- As an effective treatment for NAAION has not been conclusively found, studies looking at potential targets for the treatment of NAAION must be highlighted as only further research can achieve a breakthrough.
- A proteomics study of systemic inflammatory markers in acute and chronic NAAION patients conducted by Mesentier-Luoro et al. identified with immunoprofiling a multitude of markers in both acute and chronic NAAION patients which were significantly unique to each group when compared to the controls, with some overlap between the acute and chronic patients. Since multiple aforementioned studies on corticosteroids aiming to decrease the inflammation component seen in NAAION have not been successful as treatments, a closer, more targeted approach to blunt the inflammatory response could be a possible treatment. Candidate novel specific targets found by Mesentier-Luoro et al. most notably included Eotaxin-3, MCP-2, TPO, and TRAIL in acute NAAION patients and in chronic NAAION, IL-1α, and CXCL10 [102]. These biomarkers reveal more specifics about the systemic inflammation profile of NAAION patients and could be targeted for treatment and help treat the inflammatory component of NAAION. It is important to note the small sample size of the study and the need for a natural history study to have a longitudinal follow-up of patients and try to decrease the effect of inter-patient variability.
- In the previously mentioned study by Kumar et al. (Section 2.2.7, 4-PBA), the unfolded protein response pathway could also serve as a promising area for future research. Kumar et al. identified within those pathways increased expressions of pro-apoptotic transcriptional regulator C/EBP homologous protein (CHOP) and decreased pro-survival chaperon glucose-regulated protein 78 (GRP78) levels in both the ON and RGCs after NAAION induction in mouse models [93]. These elucidated pathways can be further studied or targeted to help further our understanding of treating NAAION.
- When investigating the effects of M01 as a neuroprotector in the aforementioned study, Chien et al. (Section 2.2.2, M01, a HECT domain-E3 ubiquitin ligase inhibitor) found that the protective effect of M01 on RGCs following ON ischemia through upregulating Nr2 was independent of the pathway they hypothesized would be involved, specifically the NEDD4 protein, as it is a known down-regulator of Nrf2. As mentioned by Chien et al., the surprising result calls for more investigation of the E3 ubiquitin ligase inhibitor pathway, or possibly another treatment that could achieve a more potent neuroprotective effect through action on NEDD4.
- Polyamidoamine Dendrimer Nanoparticles:

To target the ischemic lesion, Guo et al. investigated the use of polyamidoamine dendrimer nanoparticles, which are non-biodegradable biocompatible molecules, to target the injured cells in both rNAION and pNAION models and, therefore, acting as a drug carrier when linked to biologically active compounds [103]. They reported that following NAAION induction, Cy-5 dendrimers selectively accumulated in astrocytes and circulating macrophages and that systemic administration offered better penetration into the eye and ON than intravitreal administration [103]. The use of dendrimers to target the ischemic lesions in NAAION may provide a novel treatment route that should be investigated further.

**Table 2:** Summary table elucidating which of the discussed treatments for NAAION have been tried in human patients and their efficacy.

Pharmacotherapy	Has This Pharmacotherapy Been Used in Human Patients?	Summary
Corticosteroids	Yes [3,4,31,33,34,35]	Potential benefit in improving BCVA in the acute phase with MP. Otherwise, no clinically significant benefit improvement in outcome measures.
Triamcinolone	Yes [4]	A systematic review and meta-analysis found this drug to improve VA and VF in two studies, which had a relatively small number of cases. Larger, more comprehensive studies are needed to support this data.
EPO	Yes [34,35]	EPO administration within five days of NAAION diagnosis led to a functional and structural neuroprotective effect on the ONs at the 6-month follow-up.
G-CSF	Yes [43]	In this study, intravitreal injection of G-CSF within 2 weeks of NAAION onset resulted in a BCVA improvement at the 1-month follow-up, but this effect was not seen in the final BCVA measurement indicating the short-term effect of this drug.
Citicoline	Yes [45]	A 500 mg/day oral solution of citicoline exerted a neuro-enhancing and neuroprotective effect in a randomized pilot study that enrolled 36 NAAION patients and 20 age-matched controls. These results are promising and need to be verified with larger studies.
Trabodenoson	No	Topical trabodenoson has shown promising results in a rodent NAION model but has yet to be tried on human NAAION patients [46].
Vincamine	Yes [48]	Vincamine led to statistically significant improvement in mean deviation of the visual field and RNFL and ganglion cell complex thickness in a study with 27 NAAION patients and 15 age-matched controls. These promising results must be verified with a larger trial.
CNTF	No	The positive neuroprotective effects of this drug have been shown in an rAION model but are yet to be verified in human studies [50].
BDNF/LM22A-4	No	BDNF and LM22A-4 have promising beneficial direct and indirect effects on animal models of NAAION, but no human studies have been performed yet [51,52].
Memantine	Yes [4]	A systematic review and meta-analysis found this drug to only improve VA when analyzed as a continuous variable but not as a categorical variable. Additionally, no improvement in VF was found.
Minocycline	No	Compared to previously mentioned neuroprotective drugs, minocycline does not show promise in its ability to preserve RGC in an rNAION model [53].
Butylidenephthalide	No	While the early experimental results in an rAION model are promising, it is essential to follow the experimental evidence with human studies to validate the effect of this drug in NAAION patients [54].
Bardoxolone methyl and omaveloxolone	No	A study of these two treatments has been conducted in an rAION and revealed that out of the two drugs, bardoxolone methyl could be a potential treatment for NAAION, but this needs to be verified with human studies [55].
Prostaglandin J₂ and MAGL/COX inhibitors	No	PGJ2 has been shown to be neuroprotective in rNAION and pNAAION models only [56]. MAGL/COX inhibitors are neuroprotective in rNAION models only when used independently [57].
QPI-1007	Yes [58,59]	Phase 1 studies showed some promise for QPI-1007 in improving VA [58]; however, phase 2/3 RCTs were terminated and data showed no significant difference [59].
RPh201	Yes [60]	Phase 1 studies established the safety of RPh201 [61], and Phase 2a demonstrated non-statistically significant improvement of BCVA [60]. Completed Phase 3 study results are unpublished and cannot be assessed.
Vitamin B3	Yes, as part of a multivitamin regimen, not alone [92]	Vitamin B3 showed neuroprotective effects in rat models of NAAION [63]. Evaluated as part of a multivitamin, mineral, and carotenoid regimen in a case series where VFI improved in NAAION patients. However, bigger studies with a comparison group must be held, and vitamin B3 alone has not been assessed [92].
M01, a HECT domain-E3 ubiquitin ligase inhibitor	No	Findings in rNAION models present the modulation of HECT domain-E3 ubiquitin ligase pathways as a new approach toward the treatment of NAAION that needs to be investigated in humans [64].
Brimonidine	Yes [4,49,66,67,68]	Neuroprotective ability in animal studies [65]; however, human trials did not find significant improvement of VA or VF [4,49,66,67,68].
Progesterone	No	Progesterone showed no neuroprotective effects in models of NAAION [69].
PLGA-Icariin	No	Demonstrated a neuroprotective effect in rat NAAION models only [70].
Puerarin	No	Demonstrated a neuroprotective effect in rat NAAION models only [72].
miR-124	No	Demonstrated a neuroprotective effect in rat NAAION models only [73].
Mesenchymal stem cells	Yes [76]	A prospective, non-randomized phase II study conducted on five NAAION patients ascertained that the treatment was safe, generally well tolerated, and showed positive results, albeit in a limited number of patients. More extensive studies are needed to verify these findings.
Mesenchymal stem cell exosome	No	This treatment has yet to be tested on animal NAAION models or human NAAION patients.
Mesenchymal stem cell-derived medium	No	The efficacy of MDCM was assessed in the rAION model and was found to preserve visual function and RGC density and reduce inflammation in the ON [74]. These findings are promising but need to be supported by human studies.
Bevacizumab	Yes [78,80,81]	The evidence for this drug in human patients is mixed, but the larger studies indicate that bevacizumab is ineffective for NAAION.
Ranibizumab	No	Studies in rAION and pNAION models have reported the drug to be ineffective for NAAION [82,83].
Aflibercept	Yes [84,85,86]	Human studies have shown promising results, which need to be supported by larger studies.
Levodopa/Carbidopa	Yes [4,90,91,92,93]	Overall, there seems to be contradicting evidence within human studies; therefore, more studies must be carried out to support one conclusion.
Aspirin	Yes [49,79,90]	Aspirin was found to be ineffective as a treatment for NAAION. As a preventative measure for the development of NAAION in the second eye, aspirin was found to have mixed results but is still recommended after an episode of NAAION in patients with vasculopathic risk factors.
Platelet-rich plasma	Yes. [91]	A prospective nonrandomized controlled trial revealed that this modality is ineffective in NAAION patients when compared to controls.
Heparin-induced extracorporeal LDL/fibrinogen precipitation	Yes [4]	This modality did not improve VA as a categorical variable when assessed in a systematic review and meta-analysis.
Anticoagulants	Yes [4]	This modality was ineffective in patients with NAAION.
A multivitamin, mineral, carotenoid, and antioxidant supplement regimen	Yes [92]	Evaluated in a case series where VFI improved in NAAION patients. However, bigger studies with a comparison group must be held [92].
4-PBA	No	Demonstrated a neuroprotective effect in rat NAAION models only [93].
Bosentan	In progress [94]	Ongoing multicenter randomized controlled trial on bosentan in NAAION [94].
Omega-3 polyunsaturated fatty acid	No	Demonstrated a neuroprotective effect in rat NAAION models only [97].
Bioengineered algae oil	No	Demonstrated a neuroprotective effect in rat NAAION models only [99].
P-Selectin	No	Demonstrated a neuroprotective effect in rat NAAION models only [100].
Anti-Nogo antibody	No	Demonstrated a neuroprotective effect in rat NAAION models only [101].

## 3. Posterior Ischemic Optic Neuropathy

PION is classified based on etiology into arteritic, non-arteritic, and perioperative [8,104,105]. Table 3 provides an overview of the treatments attempted for PION subtypes. 

### 3.1. Arteritic Posterior Ischemic Optic Neuropathy

Arteritic PION is a complication of GCA, and its management follows a similar approach to A-AION, as discussed above. Treatment involves prompt and aggressive systemic steroid therapy aimed at preventing further visual loss, rather than restoring lost vision [8,104].

### 3.2. Non-Arteritic Posterior Ischemic Optic Neuropathy

Disorders other than GCA can cause non-arteritic PION. Multiple systemic diseases are associated with non-arteritic PION [104], including arteriosclerosis, atherosclerosis, arterial hypertension, diabetes mellitus [106,107], hypotension [106,108], hemodialysis [109,110], and epidural hematoma [111]. There is currently no standard treatment for non-arteritic PION. However, high-dose steroid therapy showed significant VA and VF improvement compared to untreated eyes [8,104]. This treatment is only sometimes successful [112]. Some case reports discuss additional agents to steroids, which could enhance the improvement in vision. For example, in one case, high-dose steroids were used on the first day of vision loss, and PGE1 was started the very next day. A marked improvement occurred within one day [112]. Another case of vision loss after hemodialysis and hypotension reported usage of EPO with prednisone for three days with considerable improvement in vision. They concluded that treatment with EPO within the first five days of injury is recommended, specifically in similar situations involving vision loss related to procedural-based hypotension [110].

### 3.3. Perioperative Posterior Ischemic Optic Neuropathy

Perioperative PION can happen with any surgery, but it most commonly occurs with spine surgeries due to the prone position for an extended period [113]. There is no effective treatment for improving vision after perioperative PION [114,115,116]. The primary approach is to focus on all the expected risk factors and solve them before surgery. There are multiple risk factors for perioperative ION, which may be influenced by patient-specific susceptibilities, including long duration in the prone position, excessive blood loss, hypotension, anemia [105,117], male sex, obesity, use of the Wilson frame, and lower percent colloid administration [118,119]. However, the risk factors for any given patient or procedure may vary and are likely multifactorial [117]. A case report considered using prednisolone tablets daily and showed mild improvement in vision [120]. In another report, the author used IV MP after cosmetic blepharoplasty with clinical improvement but presented with a pale optic disc and an ON-related VF defect [121] One more case report combined hyperbaric oxygen therapy and steroids successfully restoring vision after postoperative PION [122].

**Table 3:** Summary of drug treatments for posterior ischemic optic neuropathy (PION) by type, mechanism of action, and effectiveness.

Drug	Mechanism of Action	Effectiveness	Arteritic PION	Non-Arteritic PION	Perioperative PION	References
Systemic steroids (e.g., prednisone)	Reduces inflammation	Prevents further visual deterioration	✓			[8,104]
High-dose steroids	Reduces inflammation	Significant improvement in visual acuity and fields, though not always effective		✓		[8,104,112]
PGE1 and high-dose steroids	Vasodilation and neuroprotection	Marked vision improvement within one day when started early		✓		[112]
EPO with prednisone	Enhances oxygen delivery to damaged tissue	Improvement in vision when administered within five days		✓		[110]
IV methylprednisolone (MP)	Reduces inflammation	Mild visual improvement, though inconsistent			✓	[120,121]
Hyperbaric oxygen therapy with steroids	Enhances oxygenation	Successfully restored vision postoperatively			✓	[122]

### 3.4. Potential Future Treatments

Currently, there are no active trials or experiments specific to PION. However, a rat model has been developed to simulate PION using a photochemical procedure [123]. Therefore, interventions can be assessed using this model in the future.

## 4. Conclusions

As discussed, the treatment of AION and PION is a topic of active research. Our manuscript summarizes the current landscape in treating AAION, NAAION, and PION. The treatment of AAION currently consists mainly of high-dose steroid regimens, with methotrexate, tocilizumab, and abatacept being the most viable candidates for steroid-sparing therapy. These steroid-sparing strategies must be studied further in the context of preventing ocular vision loss instead of just in the treatment of GCA. As for NAAION, some pharmacotherapeutic approaches have shown promise in preliminary studies, such as animal studies and early human studies; there is still a long way to go before a specific unanimous treatment regimen is established. Similarly, despite the various treatment options explored, there is still no universally effective therapy for PION. Future research, mainly using animal models such as the photochemical rat model, offers the potential for developing new standard treatments to better manage and potentially prevent this condition.

## Abbreviations

AAION (arteritic anterior ischemic optic neuropathy); NAAION (non-arteritic ischemic optic neuropathy); PION (posterior ischemic optic neuropathy); ION (ischemic optic neuropathy); AION (anterior ischemic optic neuropathy); GCA (giant cell arteritis); SPCA (short posterior ciliary arteries); ONH (optic nerve head); ON (optic nerve); RGC (retinal ganglion cell); TNF-α (tumor necrosis factor-alpha); MP (methylprednisolone); ESR (erythrocyte sedimentation rate); CRP (C-reactive protein); IV (intravenous); IL (interleukin); CTLA-4 (cytotoxic T-lymphocyte-associated protein 4); PGE1 (prostaglandin E1); rAION (rat model of anterior ischemic optic neuropathy); PBS (phosphate-buffered saline); BCVA (best corrected visual acuity); RNFL (retinal nerve fiber layer); OCT (optical coherence tomography); VER (visual evoked response); VA (visual acuity); VF (visual field); IVTA (intravitreal triamcinolone); VEP (visual evoked potential); EPO (erythropoietin); G-CSF (granulocyte colony-stimulating factor); PERG (pattern electroretinogram); HFA MD (Humphrey 24-2 visual field mean deviation); A1R (A1 receptor); rNAION (rodent NAION model); CNTF (ciliary-derived neurotrophic factor); CFP (cyan fluorescent protein); BDNF (brain-derived neurotrophic factor); TrkB (tropomyosin-related kinase B); LM22A-4 ((N,N’,N’-tris [2-hydroxyethyl])-1,3,5-benzene tricarboxamide); BP (butylidenephthalide); RTA 402 (bardoxolone methyl); RTA 408 (omaveloxolone); NFκB 9 nuclear transcription factor nuclear factor kappa beta); PGJ2 (prostaglandin J2); PPARγ (nuclear factor peroxisomal proliferator-activated receptor-gamma); IVT (intravitreal); pNAAION (primate model of non-arteritic anterior ischemic optic neuropathy); MAGL (monoacylglycerol lipase); AA (arachidonic acid); COX1/2 (cyclooxygenase 1/2); NAD+ (nicotinamide adenine dinucleotide); NADP+ (nicotinamide adenine dinucleotide phosphate); FVEP (flash visual evoked potential); HECT (homologous E6-associated protein carboxyl terminus); NRF2 (nuclear factor erythroid 2-related factor 2); TXNIP (thioredoxin interacting protein); NLRP3 (NLR family pyrin domain containing 3); MCAO (middle cerebral artery occlusion); PLGA (poly(lactide-co-glycolide)); IKK-β (inhibitor of nuclear factor kappa-B kinase subunit beta); MSCs (mesenchymal stem cells); MDCM (MSC-derived conditioned medium); VEGF (vascular endothelial growth factor); pNAION (primate non-arteritic anterior ischemic optic neuropathy model); PRP (platelet-rich plasma); HELP (heparin-induced extracorporeal LDL/fibrinogen precipitation); 4-PBA (4-phenylbutyric acid); ER (endoplasmic reticulum); ω-3 PUFA (omega-3 polyunsaturated fatty acids); DHA (docosahexaenoic acid); EPA (eicosapentaenoic acid); CHOP (C/EBP homologous protein); GRP78 (glucose-regulated protein 78).
